# Three-dimensional optoacoustic imaging of nailfold capillaries in systemic sclerosis and its potential for disease differentiation using deep learning

**DOI:** 10.1038/s41598-020-73319-2

**Published:** 2020-10-05

**Authors:** Suhanyaa Nitkunanantharajah, Katja Haedicke, Tonia B. Moore, Joanne B. Manning, Graham Dinsdale, Michael Berks, Christopher Taylor, Mark R. Dickinson, Dominik Jüstel, Vasilis Ntziachristos, Ariane L. Herrick, Andrea K. Murray

**Affiliations:** 1 Technical University of Munich, School of Medicine, Chair of Biological Imaging, Munich, Germany; 2 Helmholtz Zentrum München, Institute of Biological and Medical Imaging, Neuherberg, Germany; 3 iThera Medical GmbH, Munich, Germany; 4 Centre for Musculoskeletal Research, Division of Musculoskeletal and Dermatological Sciences, University of Manchester, Manchester, M13 9PL UK; 5 Salford Royal NHS Foundation Trust, Manchester Academic Health Science Centre, Salford, M6 8HD UK; 6 Centre for Imaging Sciences, Division of Informatics, Imaging and Data Sciences, University of Manchester, Manchester, M13 9PL UK; 7 Department of Physics and Astronomy, University of Manchester, Manchester, M13 9PL UK; 8 Photon Science Institute, University of Manchester, Manchester, M13 9PL UK; 9 NIHR Manchester Biomedical Research Centre, University of Manchester, Manchester, M13 9PL UK

**Keywords:** Biomarkers, Medical research, Computational science, Rheumatology, Rheumatic diseases

## Abstract

The autoimmune disease systemic sclerosis (SSc) causes microvascular changes that can be easily observed cutaneously at the finger nailfold. Optoacoustic imaging (OAI), a combination of optical and ultrasound imaging, specifically raster-scanning optoacoustic mesoscopy (RSOM), offers a non-invasive high-resolution 3D visualization of capillaries allowing for a better view of microvascular changes and an extraction of volumetric measures. In this study, nailfold capillaries of patients with SSc and healthy controls are imaged and compared with each other for the first time using OAI. The nailfolds of 23 patients with SSc and 19 controls were imaged using RSOM. The acquired images were qualitatively compared to images from state-of-the-art imaging tools for SSc, dermoscopy and high magnification capillaroscopy. The vascular volume in the nailfold capillaries were computed from the RSOM images. The vascular volumes differ significantly between both cohorts (0.216 ± 0.085 mm^3^ and 0.337 ± 0.110 mm^3^; p < 0.0005). In addition, an artificial neural network was trained to automatically differentiate nailfold images from both cohorts to further assess whether OAI is sensitive enough to visualize anatomical differences in the capillaries between the two cohorts. Using transfer learning, the model classifies images with an area under the ROC curve of 0.897, and a sensitivity of 0.783 and specificity of 0.895. In conclusion, this study demonstrates the capabilities of RSOM as an imaging tool for SSc and establishes it as a modality that facilitates more in-depth studies into the disease mechanisms and progression.

## Introduction

**Figure 1:**
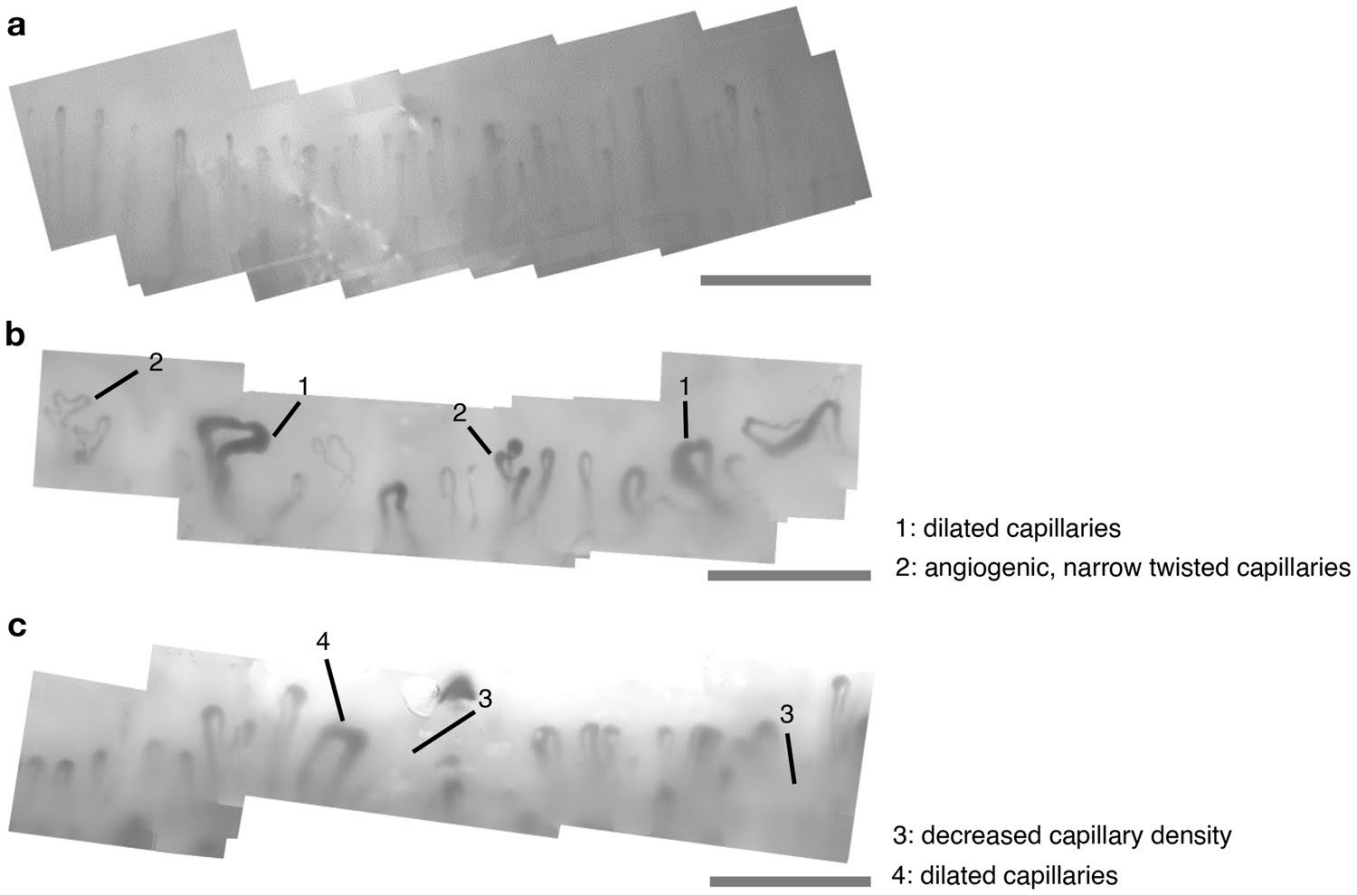
Examples of nailfold capillaries imaged with high magnification green light microscopy (optical magnification: × 200). (**a)** nailfold capillaries in a healthy control with narrow, straight, uniformly distributed capillaries and (**b,c**) vasculopathy observed at the nailbed for patients with SSc with (**b**) 1: dilated capillaries and 2: angiogenic, narrow twisted capillaries and with (**c**) 3: decreased capillary density with areas of avascularity and 4: dilated capillaries. Multiple images were stitched together to depict a larger field-of-view. All scale bars, 500 μm.

Systemic sclerosis (SSc), an autoimmune disease, causes fibrosis of the skin and internal organs as well as microvascular changes. Microvascular alteration is responsible for much of the morbidity of SSc, including Raynaud’s phenomenon and digital ulcers^1^. These microvascular changes are most easily observed in the skin at the nailfold; characteristic SSc patterns are observed as decreased capillary density, increased capillary width and angiogenesis^2,3^. As such, nailfold capillaroscopy (green or white light microscopy of the capillaries) has been used widely as a research tool for SSc. It allows easy assessment of changes in the capillary structure specific to SSc at the nailfold. Exemplary nailfold capillary patterns observed with high magnification microscopy in healthy controls in comparison to patients with SSc are illustrated in Fig. 1. Acknowledging the importance of capillary patterns in SSc, capillary abnormality was included in the American College of Rheumatology/European League Against Rheumatism 2013 classification criteria^4^ for SSc. For diagnostic purposes, capillaries only need to be visualised once, for which the 2D projective view of shallow capillaries provided by dermoscopes and higher magnification microscopy, like videocapillaroscopy, can often be sufficient. However, in research, where a deeper understanding of the disease physiology is the goal, novel and sensitive ways to visualise longitudinal and functional changes (e.g. perfusion and oxygenation) in the capillaries, in vivo, and in a three-dimensional view, are of great interest as they may offer further insights into the progression of the disease and its mechanism.

Optoacoustic (photoacoustic) imaging (OAI) is such a technique, allowing 3D visualisation and quantification of the capillaries for a better understanding of the capillary structure. OAI combines the advantages of ultrasound and optical imaging; ultrasound having good depth of penetration due to less scattering, and optical imaging taking advantage of absorption by endogenous chromophores^5,6^. Tissue is illuminated with laser light, which upon absorption, for instance by haemoglobin in blood vessels, causes thermoelastic expansions that in turn generate pressure waves that can be detected acoustically.

Several studies have previously investigated patients with SSc using OAI: Eisenbrey et al. performed functional measurements taking optoacoustic images of 7 patients with SSc and 15 healthy controls prior to and after a hand cold challenge to measure changes in oxygenation at the fingertip^7^. Reductions in oxygenation following the cold challenge were larger in the patients with SSc than in the control cohort (area under the receiver operating characteristic (ROC) curve = 0.91). Liu et al. employed multispectral optoacoustic elastic tomography, a variation of optoacoustic imaging, to measure stiffness, oxygenation and haemoglobin levels in the skin of the hands and fingers in 3 healthy controls and 3 patients with SSc^8^. Patients with SSc had increased skin stiffness and decreased oxygenation and haemoglobin levels as compared to the control group. Masthoff et al. also identified oxygenation decreases in the fingers of patients with SSc (N = 7) as compared to controls (N = 8)^9^. The relatively small number of patients included in these studies is a major limitation. While these studies mainly focused on investigating functional changes, tissue stiffness and oxygenation, OAI also has the potential to resolve microvasculature in high resolution. Using raster-scanning optoacoustic mesoscopy (RSOM)^10^, a study by Aguirre et al.^11^ reports the capability of visualizing the nailfold capillaries in 6 healthy subjects and validates the results with microscopy images by extracting and comparing features like density and width of capillaries in both modalities. However, this was a proof-of-concept study that only included healthy subjects. To our knowledge, no studies have been carried out so far studying capillary structure in SSc using OAI.

We hypothesised, that RSOM would be suitable for visualising the structural differences in the microvasculature between patients with SSc and healthy controls in 3D. Specifically, we assumed that imaging capillary patterns in the nailfolds of both groups in high resolution and 3D may, in the future, offer a means of gaining insight into the progression of SSc and its mechanisms and, thus, improve understanding of SSc pathophysiology, especially when used in longitudinal studies. For longitudinal studies to work, however, the first step is to show that a discrimination between healthy controls and subjects with SSc is possible using RSOM, thereby demonstrating sensitivity of the system to differences. Without this, structural changes over time are also unlikely to be identified. In this initial feasibility study, we imaged and analysed the nailfold capillaries in a cohort of 23 patients with SSc as compared to a control group of 19 participants with no known vascular disease using OAI for the first time. Images were acquired using RSOM and visually (qualitatively) compared to capillaroscopy images for validation. In addition to visual comparison to the gold standard, we computed and compared the vascular volumes in the nailfold of both groups based on the optoacoustic images, a volumetric feature that might offer a sensitive measure of change and severity but cannot be extracted from the 2D capillaroscopy images. Furthermore, we applied a machine learning-based approach to automatically differentiate between RSOM nailfold images of healthy subjects and patients with SSc in order to assess the sensitivity of the technique in visualizing the differences in microvasculature between cohorts. Our results establish OAI imaging as a modality not just for functional, but also structural investigation of SSc.

## Methods

### System

**Figure 2:**
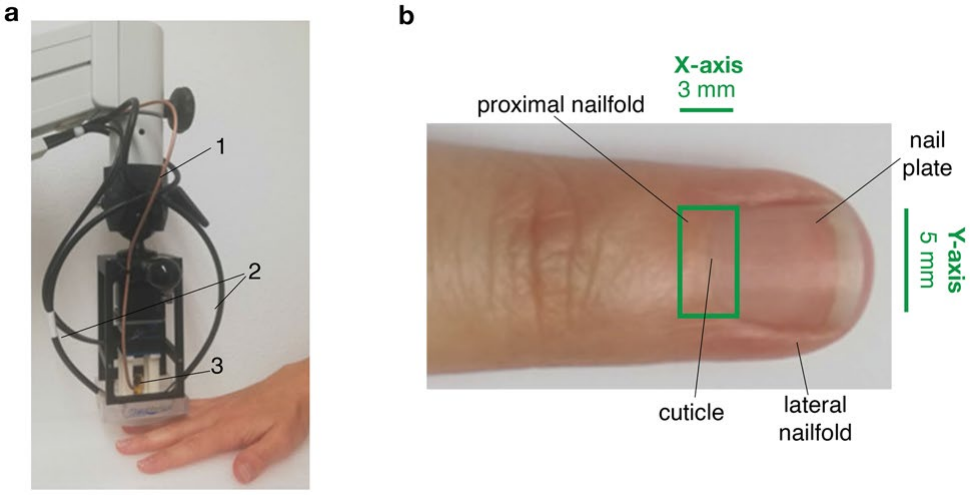
Study setup. (**a)** RSOM imaging system (RSOM Explorer C50) used to obtain OAI images in the nailfold with 1: flexible mechanical arm, 2: laser fibre optic cable, 3: 50 MHz detector. (**b)** Finger viewed from above showing approximate area imaged by OAI system (with field-of-view marked in green).

An RSOM Explorer C50 [iThera Medical GmbH, Munich, Germany], was used for imaging. All parameters for the imaging procedure were within maximal permissible exposure limits for eye and skin safety according to IEC 60825-1:2014 and EN207-2017. Furthermore, the system was equipped with a laser safety foot pedal and emergency off-button for safe operation of the laser and was used alongside appropriate laser safety goggles with suitable protection level**.** The imaging area was illuminated with a monochromatic, nanosecond pulsed, diode pumped solid state laser (532 nm, 2.5 ns pulse width, 350 Hz pulse repetition rate, 80 µJ pulse energy) located at the end of a flexible mechanical arm for optimal positioning. The laser light was delivered through a customized fibre bundle with two fibre optic cables (beam spot size: 3.1 × 2 mm) which were combined with a custom-made spherically focused LiNbO_3_ ultrasound detector (50 MHz centre frequency, 11–99 MHz bandwidth, 3 mm focal diameter, 3 mm active element distance, f-number: 1) into one scanning head. Two motorized stages enabled raster scanning over a field of view of up to 6 × 6 mm with a step size of 20 µm. An interchangeable water-filled interface unit coupled the scan head to the ultrasound gel-covered skin surface in order to optically and acoustically match the tissue probe head to the tissue. Figure 2a shows the imaging setup including the flexible mechanical arm and the scanning head. A more detailed schematic on the system setup was previously presented by Schwarz et al. (with a multispectral laser instead of a monochromatic one)^12^.

As a comparison, images were also acquired with high magnification capillaroscopy (optical magnification: × 200) [Optillia, Sollentuna, Sweden] and with dermoscopy (optical magnification: × 10) [Dermlight CA, USA].

### Study Participants

Twenty-three patients with SSc and 19 healthy controls participated in the study (demographics in Table 1). For the patient group, subjects who fulfilled the ACR/EULAR 2013 criteria for SSc^4^ [Catalogue of classification criteria published by a joint committee of the American College of Rheumatology (ACR) and European League Against Rheumatism (EULAR)] were recruited into the study. Volunteers with no known underlying vascular disease, including Raynaud’s phenomenon, diabetes and hypertension, were recruited as healthy controls. Participants were asked to refrain from caffeine and nicotine for 4 h prior to the study and to attend without creams or make-up on the area to be imaged.

**Table 1:** Demographics and clinical features of the different subject groups.

	Healthy control N = 19	SSc N = 23
**Demographics**		
Age, median [IQR] years	50 (38–55)	65 (57–69)
Female, number [%]	17 (89%)	19 (83%)
Smoking, number [%]	1 (5%)	1 (4%)
Duration of RP, median [IQR] years	N/A	18 (12–28)
Duration of SSc^a^, median [IQR] years	N/A	11 (5–18)
**Current medication, number [%]**		
Calcium channel blockers (CCB)	N/A	12 (53)
Angiotensin converting enzyme (ACE) inhibitors	N/A	4 (17)
Endothelin-I receptor antagonist	N/A	1 (4)
Angiotensin II receptor antagonist	N/A	1 (4)
Phosphodiesterase 5 inhibitors	N/A	3 (13)
Immunosupressant therapy	N/A	4 (17)
**Indicators of severe finger ischaemia, number [%]**		
IV iloprost (last 12 months)	N/A	2 (9)
Debridement	N/A	0
Amputations	N/A	0

Values are number [%] or median [interquartile range, IQR]

*RP* Raynaud’s phenomenon, cold hands with colour changes is often the first symptom of SSc. *SSc* systemic sclerosis.

^a^Defined as duration from first non-RP clinical feature.

All participants gave written informed consent. The study was approved by the South Central, Oxford B ethics committee (NRES number 17/SC/0047) and followed institutional guidelines.

### Imaging

For the study, a field of view of 3 × 5 mm on the nailfold of each ring finger was imaged with RSOM with a resolution of 40 × 40 µm in the x–y plane and 10 µm in the z-plane. Figure 2b shows the finger with the imaged area marked in green. The image acquisition took approximately 2 min per finger. Structures up to approx. 1 mm depth could be detected. The recorded data were amplified using a 63 dB low noise amplifier and subsequently reconstructed with a beam-forming algorithm, which models the sensitivity field of the focused detector. Additionally, signals were divided into two frequency sub-bands to represent larger (11–33 MHz, illustrated in red) and smaller (33–99 MHz, illustrated in green) structures, as described in detail by Omar et al. ^13^, and both frequency bands were reconstructed in the same manner. This allows for better visualization of the tissue structures, since it can prevent the fine structures in the higher frequencies from being masked by the larger structures in the lower frequencies. 3D volumetric images of all three datasets (all, high and low frequencies) were generated for further processing and analysis. For best 2D rendering, MIPs were created after the frequency bands of both high and low frequency volumes were equalized^14^ and both images were merged with the low frequency image in the red channel and the high frequency one in the green channel.

The same fingers were imaged with high magnification capillaroscopy (× 200, 2 image frames taken over the nailfold) [Optillia, Sollentuna, Sweden] and dermoscopy (× 10, single image of whole nailfold) [Dermlight, CA, USA]. A small amount of olive oil was placed on the finger for optical matching to enhance capillary visualisation with both techniques.

Images were taken between the 9th and 13th of April, 2018. Four subjects were imaged twice (after removing and repositioning the hand) to assess reproducibility.

All measurements were taken in a temperature-controlled room (23 °C) following acclimatisation.

### Image analysis

#### Qualitatitive analysis

The RSOM images of the nailfold were compared qualitatively with capillaroscopy images in order to compare the capillary structures as imaged by both techniques. It was investigated how well the different systems resolved the capillaries; whether the same capillaries were visible in all three imaging systems and whether the vascular alterations and pattern caused by SSc could be observed in RSOM images.

#### Vascular volume

The total blood volume for all RSOM nailfold images was determined using ImageJ^15^ for the quantitative comparison of patients with SSc versus the control group. The images were exported through rLabs [iThera Medical GmbH, Munich, Germany] as 32-bit images to ImageJ with a pixel scaling of 20 × 20 × 4 µm (*x, y, z* direction). All three types of datasets generated were used: one containing all frequencies acquired (11–99 MHz), one containing only low frequencies (11–33 MHz) representing larger vessels in the nailfold with diameters of around 36–112 μm and one containing only the high frequencies (33–99 MHz) representing only the ‘fine’ vasculature with vessel diameters around 12–37 μm. For the image with all frequencies, an MIP image was generated and a region of interest (ROI) was manually selected around the nailfold vessels (excluding signals from the nail). A signal intensity threshold based on the volume histogram was set to exclude background and noise signals. The thresholded volume was then quantified and summarized to calculate the total number of voxels (3D volume pixels) representing blood within the vessels. As the size of the ROI in the overall volumes might differ in different scans, the proportion of the ROI in the overall volume was computed and the total blood voxel count was divided by it to make the computed vascular volumes of all scans comparable to each other. The vascular volume of a subject was then determined as the mean value computed for both fingers.

The vascular volume between patients with SSc and healthy controls was compared using an unpaired two-tailed Student’s t-test. P-values of 0.05 or less were considered statistically significant. Reproducibility was compared with a Bland–Altman plot and measured with the intraclass correlation coefficient.

#### Machine learning based automatic differentiation

**Figure 3:**
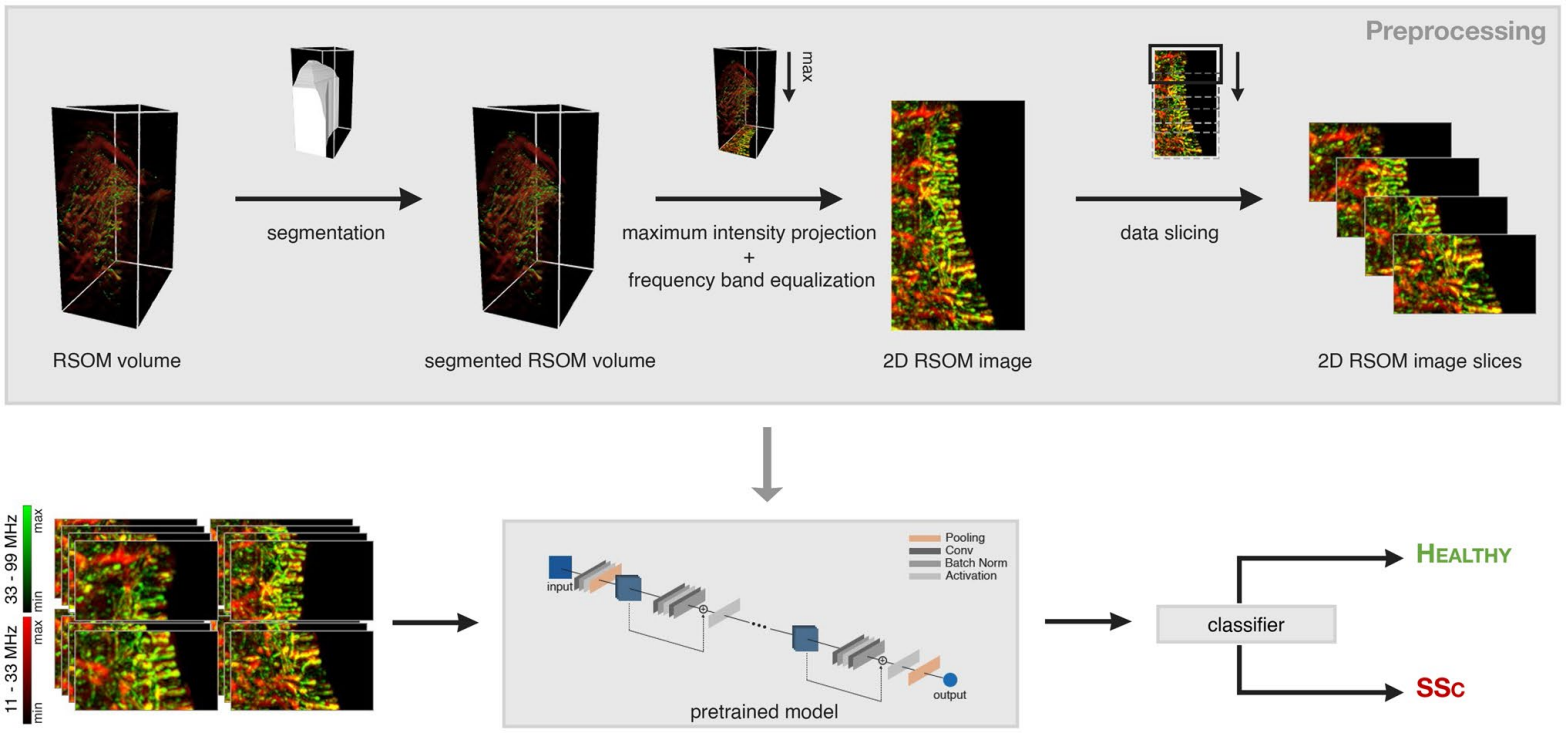
Schematic of machine learning process. RSOM volume is segmented, an MIP is created and the resulting 2D image is sliced into multiple image slices. For each image slice a pretrained deep neural network, ResNet18, predicts whether it belongs to a healthy control or a patient with SSc. The final classifier aggregates the network output for the image slices per patient and computes the prediction score for each patient. The MIPs combine the low and the high frequency bands following equalization, with the high frequency image in green and the low frequency image in red.

To assess the capabilities of RSOM for visual differentiation of patients with SSc in comparison to the control group, a machine learning model was trained for automatic disease classification. Figure 3 shows a schematic illustrating the automatic classification process. A 3D segmentation of the volume was performed by manually segmenting the nailfold region in MIP images along each dimension by hand. The ROI was then defined as the intersection of all three segmentations. Voxels outside of the ROI were ignored and therefore zeroed for further processing steps. 2D projection images of the RSOM volumes along the *z*-dimension (top view of the nailfold) were created. In order to create those images, the frequency bands of the high frequency and the low frequency volumes were equalized^14^ and the maximum intensities were projected along the *z*-dimension. Both images (MIP of high frequency volume and MIP of low frequency volume) were then merged into a single 2-channeled 2D image. Every MIP image was sliced into multiple overlapping image strips of fixed size (144 × 100 pixels) for data augmentation purposes. Due to the non-uniform occurrence of vascular changes in SSc, considering an ROI as large as possible gives the model the best starting point to learn and assess the patterns in the vasculature that are specific for each group. However, due to the small size of the study and consequently small amount of data, we decided to use multiple smaller image strips instead of one large image per patient in order to avoid overfitting. The size of the image strip was selected in a way that it was both large enough to show some local vascular patterns but still small enough to create an adequate number of image strips per patient. Instead of training a naive model from scratch, the classification was performed using transfer learning^16^ with a pre-trained neural network to avoid over-fitting due to the small amount of data. The network initially learns general image features independent of the application from a large exhaustive dataset during pre-training and is subsequently refined during training to extract more specific, task-related features. For this study, a model of the ResNet18 architecture^17^ pre-trained on the ImageNet dataset^18^ was used for the classification of patients with SSc against the control based on the RSOM image strips. During training, all the early layers were frozen and only the last two building blocks of the architecture of two convolutional layers each and the subsequent fully-connected layers were fine-tuned. Early layers of neural networks contain general and task-independent features, while later layers are found to be more specific and, therefore, less transferable^16^. Consistently, it has been found that the reuse of pretrained features mostly happens in the lowest layers and that those layers, especially in larger models, tend to change much less during training^19^. Hence, to further reduce the risk of overfitting due to our small dataset, we chose to focus our fine-tuning only on the higher-level layers. The training was performed using SGD and cross entropy loss. A detailed description of the training configurations and procedure can be found in Supplementary section B. The prediction score for a subject is then computed as the average of the network outputs for all image strips of that subject’s scans. The classification of a subject as patient with SSc or healthy control was then performed based on the prediction score using a fixed cut-off value.

The performance of the classifier was measured using leave-one-out cross validation. In each run, all image slices of one subject were held out for testing, while the model was trained and validated on the remaining data (80% for training, 20% for validation). The splitting was performed subject-wise and it was ensured that all image slices of one subject were kept within only one of the three datasets. The network accuracy was determined to measure the quality of prediction per image slice. The ROC curve was computed based on the predictions score for each subject. The optimal cut-off value was determined as the point on the ROC curve closest to the (0, 1)-point in the graph, simultaneously maximizing specificity and sensitivity of the classifier. The training framework for the machine learning approach was implemented in PyTorch.

## Results

### Qualitative analysis

**Figure 4:**
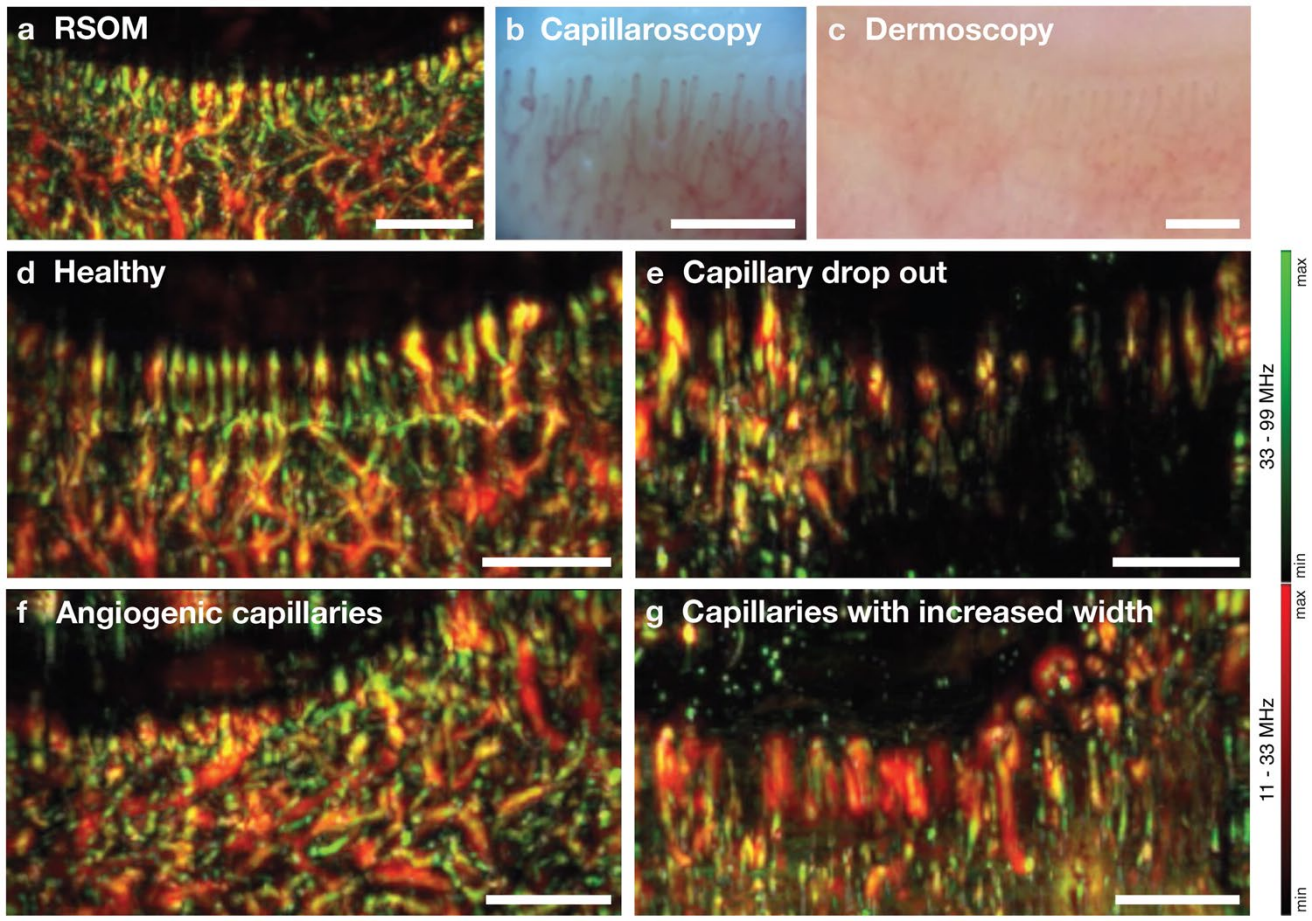
Visual Comparison. (**a)** MIP image of an RSOM volume of a healthy nailfold compared to optical imaging with (**b**) capillaroscopy and (**c**) lower magnification dermoscopy; (**d**) Examples of RSOM images taken from a healthy control with straight, uniformly distributed capillaries and (**e**–**g**) patients with SSc showing (**e**) capillary drop out, (**f**) angiogenic, twisted capillaries and (**g**) increased capillary width. The MIP images are a combination of the images from the high and low frequency bands after equalization (green: high frequencies, smaller structures, red: low frequencies, larger structures, yellow: merging of both frequency bands). All scale bars, 1 mm.

The images taken at the nailfold with the three imaging techniques were first visually compared. Figure 4a–c shows the capillaries of a healthy control as visualized by each modality. More comparisons can be found in Supplementary Fig. S1. While RSOM provides higher resolution 3D images and visualizes vasculature more clearly and with better contrast than dermoscopy, high magnification capillaroscopy resolves vessels in higher definition. By qualitatively comparing these RSOM images to those of capillaroscopy, it is possible to identify the same capillaries if images are taken at the same location. However, logistically, obtaining the identical field-of-view in the RSOM and capillaroscopy images was not always feasible, due to the small area being imaged.

Additionally, MIPs of 3D RSOM images of patients with SSc and healthy subjects were qualitatively compared to each other. Using RSOM, differences in capillary patterns could be visually identified. Figure 4d shows an RSOM image taken from a healthy control with clearly identifiable straight and uniformly distributed capillaries at the nailfold. Figure 4e–g, in contrast, illustrate the characteristic vasculopathy in SSc; demonstrating capillary loss and decreased capillary density (in Fig. 4e), angiogenic, twisted capillaries (see Fig. 4f) or dilated nailfold capillaries (see Fig. 4g).

3D videos of the RSOM volumes shown in Fig. 4 are provided in supplementary data (Supplementary Video V1 corresponding Fig. 4a and Supplementary Videos V2–V5 corresponding Fig. 4d–g).

#### Vascular volume

**Figure 5:**
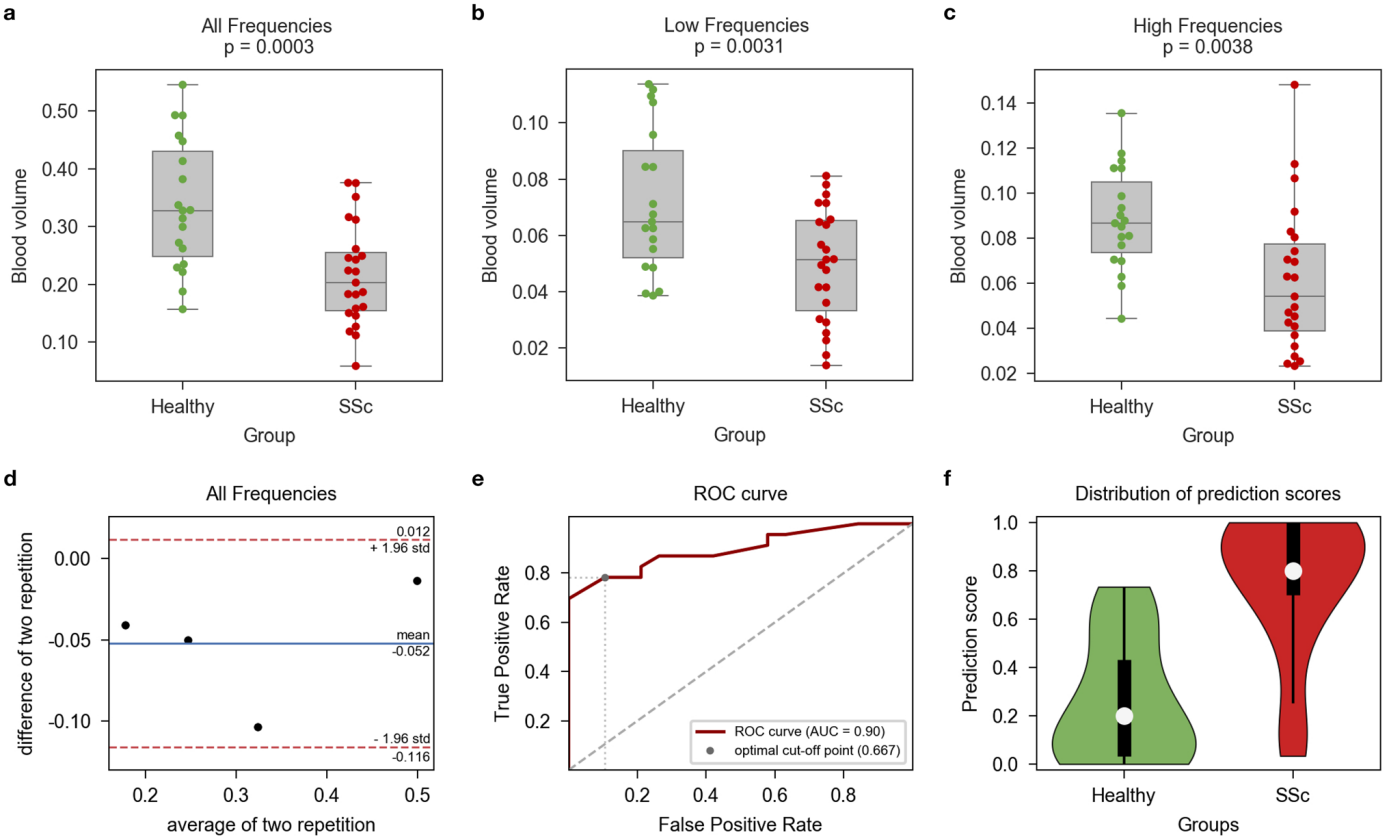
Analysis results. (**a**–**c**) Vascular volume for healthy controls vs subjects with SSc for the (**a**) all-frequency images, (**b**) low-frequency images (displaying large vessels) and (**c**) high-frequency images (displaying small vessels); the boxes represent the inter-quartile range while the whiskers extend to show the whole range of the distribution. (**d**) Bland–Altman plot showing reproducibility of vascular volume as a feature computed in RSOM (containing all frequencies); (**e**) ROC-curve for machine learning-based differentiation of RSOM images; (**f**) Distribution of prediction scores from machine learning-based classification.

Quantitative analysis of the vascular volume in the RSOM volumes for the full frequency range and for the subsets of frequencies representing the different vascular structure sizes is shown in Fig. 5a–c. For all frequencies the vascular volumes [mean ± standard deviation] between groups was 0.216 ± 0.085 mm^3^ (patients with SSc) and 0.337 ± 0.110 mm^3^ (healthy controls); p < 0.0005. For the smaller frequency threshold (larger structures), the volumes were 0.050 ± 0.020 mm^3^ (patients with SSc) and 0.072 ± 0.025 mm^3^ (controls); p = 0.0031. For the higher frequency (fine structures) the volumes were 0.061 ± 0.031 mm^3^ (patients with SSc) and 0.088 ± 0.022 mm^3^ (controls); p = 0.0037.

Four subjects were imaged twice using RSOM in order to determine the reproducibility of the computation of vascular volume. The Bland–Altman plot in Fig. 5 d demonstrates the reproducibility of the vascular volume feature based on RSOM images containing the full frequency range. The differences between initial and repeat measurement of all four subjects lay within the limits of agreement (μ ± 1.96 * σ) with an intra-class correlation coefficient of 0.902, which implies good reproducibility. However, we acknowledge the small number of observations in this calculation.

### Machine learning based automatic differentiation

The purely image-based automatic classification of RSOM images into patients with SSc and healthy controls was performed by our pretrained neural network with an accuracy of 0.749 on single image strips. However, since vascular changes in SSc are not uniform, and therefore may not be apparent in all image slices, looking at all image strips of a subject in their entirety provides a better picture of the status of capillary patterns. Thus, the prediction scores, which are the averaged prediction for all image slices per participant, depict the classifier’s performance in a better way. Figure 5e shows the ROC curve of our classification model [Area Under the ROC Curve = 0.897]. Figure 5f visualizes the distribution of prediction scores in each group (prediction score: 1 = SSc and 0 = healthy). For the prediction score, 0.667 was identified as the optimal cut-off value to determine whether a subject should be classified as a patient with SSc or healthy control. Using this cut-off for the subjects’ prediction scores, the trained classifier achieved an overall balanced accuracy of 0.848 in differentiating both groups, with a sensitivity of 0.783 and specificity of 0.895.

## Discussion

In SSc, imaging the characteristic vasculopathy is key both to diagnosis and to elucidating pathophysiology, monitoring disease progression and response to treatment. RSOM offers an approach to visualize fine microvasculature in 3D and in high resolution. We have successfully demonstrated that using OAI, RSOM in particular, it is both possible to visualise SSc-specific vasculopathy in the capillaries of the nailfold and to measure significant differences in vascular volume between the images from patients with SSc and those from a control cohort. The machine learning-based analysis demonstrated that, despite the small number of subjects, it is possible to automatically differentiate between patients with SSc and healthy controls based solely on OA images of the nailfold, with high sensitivity (78.3%) and specificity (89.5%).

RSOM measurements performed in our study show good reproducibility. It has been previously reported that the reproducibility of quantitative measurements (based on capillaroscopy) in SSc relies on repeated measurements being taken at the same site due to the vascular heterogeneity that occurs at the nailfold^20^. When performing repeated measurements with RSOM but also when comparing RSOM to capillaroscopy in our study, logistically, it was not always possible to image the exact same field-of-view and observe the same capillary pattern in both images (see Supplementary Fig. S1) demonstrating the difficulty in replicating measurements. Despite the logistical difficulties, the reproducibility of the quantitative measurement taken with RSOM was good.

RSOM is complimentary to nailfold capillaroscopy both from a diagnostic and a research perspective. Capillaroscopy is the state-of-the-art clinical diagnostic technique for observing nailfold capillaries. It can be used diagnostically at lower magnifications, cheaply and portably. However, in certain subjects for reasons that are not fully understood, it can be difficult to observe capillaries clearly. In such borderline cases, RSOM could complement nailfold capillaroscopy to get a clearer view of the vasculature. Also, when used to elucidate the pathophysiology of the SSc vasculopathy, capillaroscopy has limitations. To monitor SSc progression, capillaroscopy is used to determine capillary density and width at the distal row in the nailfold. However, identifying which capillaries lie in the distal row based on a 2D capillaroscopy image has been a difficult and contentious task to date. RSOM allows 3D imaging and increases the understanding of vascular structures. The additional dimension helps to unambiguously identify distal row capillaries and distinguish them from other capillaries, which might otherwise be projected to similar locations in 2D images. Furthermore, capillaroscopy is limited to the plane of the skin surface. When carrying out quantitative measurements of the vessels across the nailfold (particularly width and tortuosity), based on the 2D image, it is not possible to identify in which plane a particular capillary is oriented. However, with RSOM these capillary measurements can be performed and quantified easily for further in-depth studies of SSc vasculopathy. Thus, RSOM may increase the usefulness and accuracy of capillaroscopy by aiding our understanding of the peripheral capillary layering structure. Moreover, thanks to its 3D capabilities, it allows the extraction of volumetric features, like vascular volume as demonstrated in this study, which may allow to assess severity and vascular changes with a higher sensitivity in the future.

Since RSOM offers the opportunity to image the capillary loops in 3D at any site of the skin, it can be used for a wider range of studies investigating not only mechanisms affecting nailfold vascularisation but whether these changes are also present in other areas of skin. In patients with SSc, it can also be applied for imaging digital ulceration, skin microcirculation at the site of calcinosis or furthering understanding of how telangiectasias form, all of which may then increase understanding and facilitate new treatments.

In addition, OAI potentially allows real-time oxygenation (by adding more wavelengths to unmix oxygenated from deoxygenated blood) and perfusion to be measured, which offer unique potential to image and understand treatment response mechanisms in real-time. It is also possible that oxygenation measures may allow increased stratification of those patients at risk of digital ulcers or other disease progression, which may in the future mean that imaging would become relevant early in the disease process as a prognostic factor or to initiate early disease intervention.

We have shown that RSOM is able to image capillaries at the nailfold in both patients with SSc and controls. Vascular volumes differ significantly between the groups and using machine learning we are able to use the characteristic vascular changes observed in SSc to discriminate between groups with high specificity and sensitivity, demonstrating the sensitivity of the modality to differences. Further investigations need to be done with regards to classification and grading of disease progression based on RSOM. Previously, it has been reported that higher magnification imaging, when comparing capillaroscopy to lower magnification optical dermoscopy, makes images more classifiable and leads to clinicians grading images more ‘severely’^21^. This may potentially impact any future grading of images from the RSOM systems as well given their different resolutions. Furthermore, a study with a larger cohort of subjects, with patients at different stages of the disease can give more profound insights on the capabilities of OAI for differentiating certain patient groups. Using OAI in larger longitudinal studies in patients will allow a deeper understanding of the vascular pathophysiology associated with SSc, as well as how it is affected by disease progression.

## Supplementary information

Supplementary Information.

Supplementary Video 1.

Supplementary Video 2.

Supplementary Video 3.

Supplementary Video 4.

Supplementary Video 5.

## Abbreviations

SSc: Systemic sclerosis
RSOM: Raster-scanning optoacoustic mesoscopy
OAI: Optoacoustic imaging
ROI: Region of interest
ROC curve: Receiver operating characteristic curve
