# Supplementary material for: Three-dimensional optoacoustic imaging of nailfold capillaries in systemic sclerosis and its potential for disease differentiation using deep learning
Source: Sci Rep. 2020 Oct 5;10:16444. doi: 10.1038/s41598-020-73319-2 (PMC7536218; doi:10.1038/s41598-020-73319-2)
Supplement: Supplementary file 1 — Supplementary Information. [file 41598_2020_73319_MOESM1_ESM.pdf]

Three-dimensional optoacoustic imaging of nailfold capillaries in systemic sclerosis and its potential for disease differentiation using deep learning.

Supplementary Information

Suhanyaa Nitkunanantharajah, Katja Haedicke, Tonia B. Moore, Joanne B. Manning, Graham Dinsdale, Michael Berks, Christopher Taylor, Mark R. Dickinson, Dominik Jüstel, Vasilis Ntziachristos, Ariane L. Herrick, Andrea K. Murray

A. Visual Comparison of RSOM, Dermoscopy and Nailfold Capillaroscopy

Supplementary Figure S1: Comparisons between images taken with RSOM, dermoscopy (optical magnification: x10) and nailfold capillaroscopy (optical magnification: x200). *Boxes highlight the position of the same areas between the imaging techniques (roman numerals match the relevant boxes). Examples are shown for comparison of* ***a)*** *healthy control capillaries;* ***b)*** *haemorrhaging;* ***c)*** *avascularity;* ***d)*** *enlarged capillaries; and* ***e)*** *tortuous capillaies. Orange circles in b) indicates a feature that can be identified in all the or most of the images. All scale bars, 500 µm.*

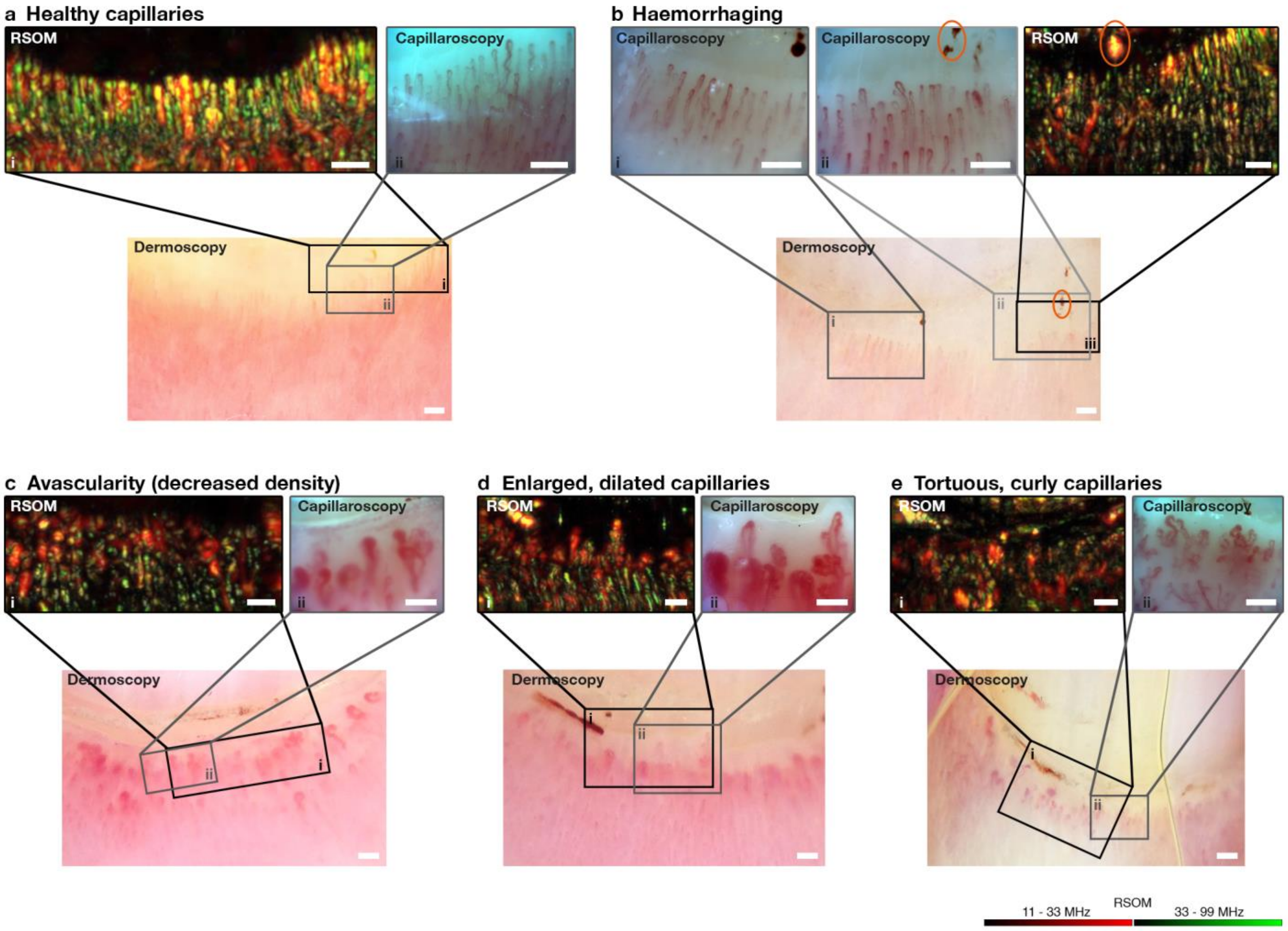

B. Training Configuration

A model of ResNet18 architecture, as described in [1], was used as base architecture for training. The model was pretrained on the 1000-class ImageNet dataset. Subsequently, the final fully-connected layer was replaced by a 2-way fully-connected layer. During training with RSOM image slices, all layers were frozen and only the last two blocks of the architecture with two convolutional layers each (each convolution followed by batch normalization and activation) as well as the fully-connected layer were fine-tuned.

The training was performed using data of 42 subjects, with 30 image slices of size 144 x 100 per patient (overall: 1260 image slices). The model was trained using leave-one-out cross validation, using all image slices of one patient for testing in each run. From the remaining data, 80% was used for training and 20% for validation – in each run, image slices of overall 33 subjects were used for training, 8 for validation and one for testing. To obtain balanced groups of image slices of healthy controls and patients with SSc within training and validation sets, some image slices were oversampled.

We trained the model using SGD with a mini-batch size of 32 and a learning rate of 0.01 (momentum = 0.9) and used cross entropy loss. The model was trained for 150 epochs, unless the classification accuracy on the validation set did not improve for more than 12 epochs, in which case the training was interrupted early.

References

1. He, K., Zhang, X., Ren, S. & Sun, J. Deep residual learning for image recognition. in *Proceedings of the IEEE Computer Society Conference on Computer Vision and Pattern Recognition* 770–778 (2016).

Supplementary Video V1. Three-dimensional nailfold visualization of a healthy nailfold by RSOM. 3D data corresponds to the MIP image in 4 a.

Supplementary Video V2. Three-dimensional nailfold visualization by RSOM taken from a healthy control with straight, uniformly distributed capillaries. 3D data corresponds to the MIP image shown in 4 d.

Supplementary Video V3. Three-dimensional nailfold visualization by RSOM taken from a patient with SSc showing capillary dropout. 3D data corresponds to the MIP image in 4 e.

Supplementary Video V4. Three-dimensional nailfold visualization by RSOM taken from a patient with SSc showing angiogenic capillaries. 3D data corresponds to the MIP image in 4 f.

Supplementary Video V5. Three-dimensional nailfold visualization by RSOM taken from a patient with SSc showing capillaries with increased width. 3D data corresponds to the MIP image in 4 g.
